# Contact Allergy in Atopic Dermatitis and Psoriasis: A Retrospective Study

**DOI:** 10.3390/diagnostics15060766

**Published:** 2025-03-19

**Authors:** Domenico Bonamonte, Aurora De Marco, Giulia Ciccarese, Paolo Romita, Giulio Giancaspro, Francesca Ambrogio, Caterina Foti

**Affiliations:** 1 Section of Dermatology and Venereology, Department of Precision and Regenerative Medicine and Jonian Area, University of Bari “Aldo Moro”, 70124 Bari, Italy; domenico.bonamonte@uniba.it (D.B.); paolo.romita@uniba.it (P.R.); giulio-giancaspro@libero.it (G.G.); dottambrogiofrancesca@gmail.com (F.A.); caterina.foti@uniba.it (C.F.); 2 Section of Dermatology, Department of Medical and Surgical Sciences, University of Foggia, Viale Pinto, 71122 Foggia, Italy; giulia.ciccarese@unifg.it

**Keywords:** contact allergy, allergic contact dermatitis, psoriasis, atopic dermatitis, patch test

## Abstract

**Background/Objectives**: The correlation between contact allergy (CA), atopic dermatitis (AD) and psoriasis is still debated. Therefore, the present study aims to retrospectively analyze the frequency of contact sensitization among patients with psoriasis and AD compared to controls, in order to further investigate the relationship between CA and the underlying immunological background. **Methods**: All data concerning patients who underwent patch testing from 2016 to 2022 in the dermatology clinic of a tertiary center in Southern Italy have been retrospectively collected. Only patients who underwent patch testing with the S.I.D.A.PA. standard series have been selected and divided into three groups: AD group, psoriasis group and control group. Acquired data were organized into database and underwent statistical examination. **Results**: A total of 2287 patients have been enrolled, including 377 AD patients, 127 psoriatic patients and 1783 controls. The most frequent allergens were nickel and balsam of Peru. Methylisothiazolinone (4.2% vs. 2.2%), paraben mix (0.3% vs. 0%) and neomycin (1.3% vs. 0.4%) significantly provided more positive reactions (PSR) in the AD group compared to the control one, and fragrance mix II displayed a higher rate of positivity in the atopic group compared to the psoriatic one (3.2% vs. 0%). **Conclusions**: Psoriasis turned out to be a possible protective factor for CA (odds ratio = 0.6), while AD seems to facilitate its development (odds ratio: 1.42). The limitations of this study mainly rely upon its retrospective nature which limited the acquisition of clinical relevance for PSR. Further studies are required to better investigate this topic.

## 1. Introduction

### 1.1. Contact Dermatitis and Allergic Contact Dermatitis

Contact dermatitis (CD) is an inflammatory cutaneous condition caused by exposure to an exogenous substance. CD is a common reason for dermatology consultations, representing 4–7% of all dermatological diagnoses annually at any age and in both genders, with a female preponderance [1]. Occupation is the leading risk factor: hairdressers, food handlers, health care workers and building and metal workers are at risk of developing CD because of repeated exposure to several common allergens [2].

The spectrum of CD includes two disease subgroups that differ in clinical and pathophysiological aspects: irritant contact dermatitis (ICD) and allergic contact dermatitis (ACD) [1,2,3,4].

ICD is the most common form of CD, representing the cutaneous response to the toxic effects of various physical or chemical environmental agents.

At the same time, allergic contact dermatitis (ACD) is an inflammatory skin reaction caused by a type IV hypersensitivity mechanism that manifests after repetitive exposure to a contact allergen in previously sensitized patients [1,3,4]. More specifically, the pathogenetic mechanism comprises two phases. The first (sensitization or afferent) phase happens when a foreign substance (allergen) penetrates the skin and binds to cutaneous proteins, determining the formation of an antigen complex [1]. This process triggers an inflammatory reaction with innate immune system activation through keratinocyte release of cytokines, such as IL-1α, IL-1β, tumor necrosis factor-α (TNF-α), IL-8, IL-18 and granulocyte-macrophage colony-stimulating factor [1,5]. Allergens are picked up by antigen-presenting cells (APCs), such as Langerhans cells, and migrate to the regional lymph nodes, where antigen-specific T helper (Th1, Th2 and Th17) and T regulatory cells are activated and then proliferate and circulate in the blood. The activation of the naïve T cells also induces the formation of memory T cells, which, during sensitization, preferentially accumulate at the allergen contact site [1,5].

In the second (elicitation or efferent) phase of the ACD pathogenesis, after re-exposure to the allergen, the allergen-specific memory T cells recognize the foreign substance and activate an inflammatory response mediated by several cytokines (interferon γ (IFN-γ), IL-2 and IL-17), leading to the development of an inflammatory infiltrate responsible for the clinical features of ACD [1]. Therefore, the memory T cells (both tissue-resident and circulating T cells) cause a rapid and intense response to any new allergen exposure, playing a key role in the recurrence of ACD and its chronicity [2,5].

The clinical features of ACD are polymorphous, depending on the chemical characteristics of the culprit substance, the type and way of exposition, and the affected body area. The skin lesions of ACD appear about 5–7 days after first contact with the allergen, while subsequent contacts need about 1–2 days to induce a skin reaction [4]. The acute phase is characterized by bright red erythema with edema, vesicles and bullae associated with intense itch. The cutaneous lesions initially involve only the area originally in contact with the allergen and then spread to distant sites by inadvertent contact or autosensitization [4]. Unlike in the ICD, the borders of the lesions of ACD are ill-defined [6]. Vesicles are fragile and tend to rupture due to their superficial localization in the epidermis, resulting in multiple confluent erosions. The chronic phase of ACD, due to the persistence of the culprit agent, is characterized by lichenification, scaling or fissured dermatitis, with or without accompanying papule-vesicles. Any area of the body can be affected and the eruption may be also generalized. The most common allergen responsible for ACD is nickel sulfate, followed by methylisothiazolinone, fragrance mix, formaldehyde and *p*-phenylenediamine [7]. However, ACD may be due to a significant number of other substances included in a wide range of everyday products, such as personal care products (even when labeled as “natural”), topical drugs and everyday household products [8,9]. A detailed medical history, an accurate physical examination and allergy diagnostic tests (patch tests) are always required to diagnose ACD, given its similarity with other inflammatory dermatoses. ACD is, therefore, identified by a positive patch test reaction (that may or may not be of current clinical relevance), while ICD is a diagnosis of exclusion [10].

The correlation of ACD with the most common chronic-relapsing inflammatory skin diseases, mainly atopic dermatitis (AD) and psoriasis, is still debated, as controversial and often inconsistent literature data are currently available.

### 1.2. Atopic Dermatitis

AD is a chronic skin disease with a lifetime prevalence of 20% worldwide [11]. It is caused by genetic predisposition, skin-barrier disruption, immune factors and environmental exposure to exogenous insults [12]. Signs and symptoms of AD include pruritus, skin pain, xerosis, oozing in acute lesions, lichenification and prurigo-like nodules in chronic lesions [12]. The distribution of the lesions may be influenced by several aspects, such as patients’ age and Fitzpatrick skin types [11]. Facial dermatitis occurs in infants, extensor dermatitis in small children, flexural dermatitis in older children and adolescents (especially in case of Fitzpatrick skin types I-III) and facial and hand dermatitis in adults [11].

The diagnosis of AD is clinically based on patient history and physical examination. Several diagnostic criteria are available: the 1980 Hanifin and Rajka criteria, the UK Working Party’s Diagnostic Criteria, and the American Academy of Dermatology criteria [12]. However, given the highly heterogeneous presentation of AD, diagnosing it with certainty may sometimes be challenging, particularly in adults, requiring a histological confirmation.

As for AD’s immunological background, two major subtypes are currently recognized (extrinsic and intrinsic), both showing Th2 signaling [13]. Extrinsic AD (about 80% of cases) is characterized by a Th2-dominant immune response, increased eosinophils and IgE levels in serum and early onset. This subtype is linked to impaired skin barrier function, high transepidermal water loss and filaggrin gene mutations, which increase the risk of developing eczema and food allergies [13].

Conversely, intrinsic AD is characterized by a Th1 and Th17 immune response with high levels of IFNγ-producing Th1 cells in the blood and normal serum IgE levels. This subtype of AD has a later onset, is often more severe and not typically related to barrier dysfunction compared to the extrinsic subtype [13].

Notably, several key pathogenetic features of AD may predispose patients to ACD: i. the intrinsic skin barrier defects, facilitating not only the acquisition of infections [14] but also the penetration of external allergens, ii. the cutaneous immune dysregulation including shared cytokine pathways and iii. the frequent use of emollients and topical medications.

Although some systematic reviews have demonstrated that ACD is a significant clinical issue in patients with AD [15,16], it is still unclear whether AD may favor or not the development of ACD [17,18,19]. Many aspects seem to support the idea that AD may be a risk factor for ACD, as AD skin shows a two-fold increased absorption rate of many different chemicals, both irritant and allergenic, possibly due to its intrinsic atopic barrier dysfunction [20]. Moreover, according to general treatment recommendations, AD patients are often prone to use topical drugs and moisturizers, which may contain possible contact sensitizers even when labeled as “hypoallergenic” [21]. On the other hand, different studies seem to support the idea that AD may even play a protective role against the development of ACD [22,23]. According to this hypothesis, as the atopic phenotype is driven by a preferential Th2-type response, the development of a Th1/T-cytotoxic (Tc)1 response, as in the case of ACD, is unlikely to occur [19].

### 1.3. Psoriasis

As for psoriasis, even less conclusive literature data regarding its exact relationship with ACD are available.

Psoriasis is a common, chronic, inflammatory skin disease with a prevalence estimated at 0.5–11.4% of adults worldwide and an age of onset with a bimodal distribution: a first peak between the ages of 16 and 22 years and a second between the ages of 57 and 60 [12]. Well-defined, erythematous plaques with overlying scales typically characterize psoriasis [12]. Clinical subtypes include chronic plaque psoriasis, inverse psoriasis, guttate psoriasis, pustular psoriasis, erythrodermic psoriasis and palmoplantar psoriasis. Regarding etiology, psoriasis manifestations are caused by accelerated epidermal proliferation in patients with a genetic predisposition [12]. The abnormal keratinocyte differentiation is determined by an immune dysregulation of innate and adaptive immune systems involving dendritic cells, T-lymphocytes, and cytokines [12]. Dendritic cells secrete IL-12 and IL-23, which activate Th proliferation, especially the Th1 and Th17 subtypes. Th1 and Th17 release inflammatory cytokines such as TNF, IL-6, and IL-17. IL-17 augments the recruitment of neutrophils in the dermis and dilates the dermal blood vessels, which causes the classic erythema of psoriatic lesions [12]. Well-known triggers of psoriasis include stress, infection, hypocalcemia, and medications, such as lithium or non-steroidal anti-inflammatory drugs [12]. Palmoplantar psoriasis has been associated with environmental factors like smoking and mechanical friction or trauma [12]. The diagnosis of psoriasis is clinical; however, biopsies may be performed to confirm the diagnosis, particularly in cases with uncertain etiology, such as erythroderma. The differential diagnosis of psoriasis comprises many dermatoses, including AD, ICD and ACD [12].

Former literature data demonstrated an inverse correlation between ACD and psoriasis [24], and even more recent works registered a lower rate of contact sensitization among psoriatic patients compared to controls [25]. Moreover, many recent findings suggest that the lower sensitization ratio registered among psoriatic patients could be due to a sort of interference of the underlying psoriatic autoimmune disorder towards the development of ACD itself, as also encountered in other systemic autoimmune diseases [26]. On the other hand, not only different studies suggested that psoriatic patients share the same sensitization rate as healthy controls [27], but it even seems that psoriatic patients may be exposed to an increasing risk of developing ACD [28].

### 1.4. The Link Between ACD, AD and Psoriasis

**Figure 1:**
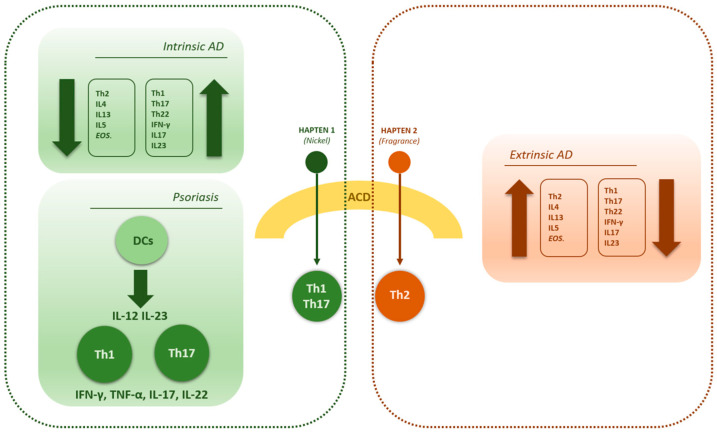
Schematic representation of possible correlations between allergic contact dermatitis (ACD), psoriasis and both intrinsic and extrinsic atopic dermatitis (AD). Colored inner boxes illustrate the most relevant immune-pathogenetic aspects of psoriasis and AD (both intrinsic and extrinsic); external dashed frames indicate possible immunological similarities and correlations between intrinsic AD, psoriasis and ACD on one hand, and between extrinsic AD and ACD on the other hand. Different colors (green and red) are used to highlight the pivotal role of the hapten’s nature (e.g., nickel or fragrances) in determining the immune-polarization of ACD response (EOS. = eosinophils; DCs = dendritic cells).

The debate linking AD and/or psoriasis to ACD recently became even more interesting, as ACD transcriptome studies on human skin demonstrated distinct polarizations of the immune response towards three of the most frequent allergens, that is to say nickel, fragrances and rubber [29]. According to this study, indeed, nickel may induce a Th1/Th17-driven response, whereas fragrances and rubber seem to polarize the immune system towards a Th2 cutaneous response, with a smaller involvement of Th1/Th17 [29]. Moreover, later literature demonstrated a reduced contact sensitization rate to thiuram mix, fragrance mix II and lanolin alcohols among patients with psoriasis compared to controls [30]. Interestingly, the immune response triggered by these allergens differs significantly from the inflammatory cytokines involved in psoriasis, while it could be way more similar to the one induced in atopic patients, as former studies showed elevated sensitization rates towards these allergens in AD, which is dominated by a Th2-polarized immune response (Figure 1) [30,31].

Therefore, the present study aims to retrospectively analyze the frequency of contact sensitization among patients with psoriasis and AD compared to controls to investigate further the relationship between contact allergy (CA) and the underlying immunological background.

## 2. Materials and Methods

The present study has been approved by the local Ethics committee and has been conducted according to the Helsinki Declaration (study number: 7649; approval protocol number: 0044455/11 May 2023). Patients selected for the study have signed written formal consent to the medical procedure at the time of the test and retrospective access to collected data has been guaranteed throughout Data Protection Impact Assessment.

All data concerning patients who underwent patch testing, according to normal clinical practice, from 2016 to 2022 in the dermatology clinic of the Policlinico of Bari were retrospectively collected. Only patients who underwent patch testing with the S.I.D.A.PA. (Società Italiana di Dermatologia Allergologica Professionale e Ambientale) standard series were selected (according to testing year: Euromedical, Calolziocorte, Italy; FIRMADiagent, Florence; ItalySmartPractice, Rome, Italy), while patients who underwent patch testing with personal products or other integrative series were excluded (Table S1).

Patch tests were applied on the back and left in occlusion for 2 days, using the Al Test (Euromedical) on Scanpor Tape (Norgesplaster, Vennesla, Norway) or the Haye’s Test Chambers (Haye’s Service, Alphen aan den Rijn, The Netherlands) on Soffix tape (Artsana, Grandate, Italy), according to the current year of the test. Readings were performed on day (D) 2, D4 and D7 according to guidelines and only confirmed results on D4 and D7 were included in the study [32].

Clinical data collection and patients’ medical histories led to the establishment of three groups: an AD group (including patients suffering from AD), a psoriasis group (including psoriatic patients) and a control group (involving patients not suffering from AD or psoriasis).

As for the AD and psoriasis groups, only patients undergoing topical therapies were enrolled in the study. Moreover, due to the study’s retrospective nature, no clinical data regarding disease severity and extension were available for AD and psoriasis patients.

Therefore, collected data underwent statistical examination. The two-sample proportion zeta test has been used to compare relative frequencies of positive skin reactions (PSR) to each tested substance among the three examined groups (statistical significance when *p*-value < 0.005). In the end, the odds ratio was calculated in order to assess a possible cause–effect correlation between AD and/or psoriasis and the development of CA (statistical significance when *p*-value < 0.05).

## 3. Results

**Figure 2:**
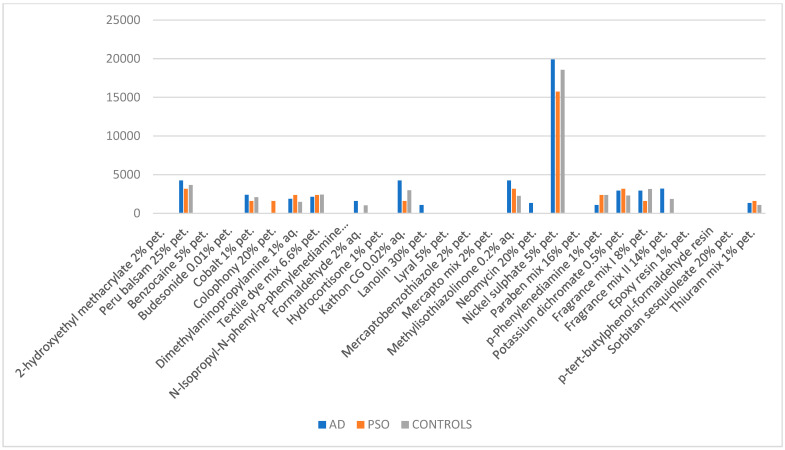
Graphical representation of relative frequencies of positive skin reactions (PSRs) for each tested substance in the three examined groups (AD = atopic dermatitis, PSO = psoriasis); data extracted from Table 1.

A total of 2287 patients (F = 1440, M = 847), ranging from 6 to 98 years old (mean age: 51.14), were enrolled in the study, including 377 patients suffering from AD (F = 250, M = 127; mean age: 40), 127 patients suffering from psoriasis (F = 65, M = 62; mean age: 57) and 1783 controls, suffering neither from AD nor from psoriasis (F = 1125, M = 658; mean age: 56). A total of 28 substances belonging to the S.I.D.A.P.A. standard series (years 2016–2022) were included in the study (Table S1). PSR occurred in 1167 cases out of 2287 (51.02%), displaying a higher relative frequency in the AD group compared to both the psoriasis group (58.35% vs. 41.73%; *p*-value = 0.00830354) and the control group (58.35% vs. 50.14%; *p*-value = 0.00373272) (Table 1, Table 2 and Table 3; Figure 2).

In all examined groups, the haptens most frequently associated with PSRs were nickel (19.9% in the AD group, 15.7% in the psoriasis group and 18.6% in the control group) and balsam of Peru (4.2% in the AD group, 3.2% in the psoriasis group and 3.6% in the control group); followed by Kathon CG (4.2%), methylisothiazolinone (4.2%), fragrance mix I and II (6.1%) and potassium dichromate (2.9%) in the AD group; methylisothiazolinone (4.2%), potassium dichromate (3.1%), dimethylaminopropylamine (2.4%), textile dye mix (2.4%) and *p*-phenylendiamine (2.4%) in the psoriasis group and by fragrance mix I (3.1%), Kathon CG (2.9%), textile dye mix (2.4%), *p*-phenylendiamine (2.4%) and potassium dichromate (2.3%) in the control group. Moreover, as for the relative frequencies of PSRs to the examined haptens, no statistically significant differences were detected between each study group (AD and psoriasis) and the control group, except for methylisothiazolinone (4.2% vs. 2.2%; *p*-value = 0.03), the paraben mix (0.3% vs. 0%; *p*-value = 0.03) and neomycin (1.3% vs. 0.4%; *p*-value = 0.045), which displayed a higher percentage of positive reactions in the AD group compared with controls (Table 2 and Table 4).

On the other hand, AD patients displayed a higher rate of PSRs to fragrance mix II compared to psoriasis patients (3.2% vs. 0%; *p*-value = 0.04), while no other statistically significant differences were found between the two study groups (Table 3).

Interestingly, psoriasis patients did not develop any PSRs to 14 tested allergens out of 28 (50%), compared to AD patients (0% PSR in the case of three tested substances out of 28; 10.7%) and control patients (0% PSR in the case of one tested substance out of 28; 3.6%).

## 4. Discussion

### 4.1. General Findings

In the present study, AD patients demonstrated a higher rate of PSRs compared to both psoriasis patients and control patients, respectively (58.35% vs. 41.73% vs. 50.14%; *p*-value = 0.00830354).

As AD patients displayed a higher rate of PSRs compared to psoriasis patients, it is possible to suggest that AD may be a risk factor for the development of skin contact sensitization (odds ratio: 1.42; *p*-value = 0.002), while psoriasis may even have a protective effect (odds ratio = 0.6; *p*-value = 0.03).

Interestingly, no differences were noticed in the development of PSRs to the tested substances in all groups, with only a few exceptions. As a matter of fact, methylisothiazolinone (4.2% vs. 2.2%; *p*-value = 0.03), the paraben mix (0.3% vs. 0%; *p*-value = 0.03) and neomycin (1.3% vs. 0.4%; *p*-value = 0.045) significantly provided more PSRs in the AD group compared to the control group, and fragrance mix II displayed a higher rate of PSRs in the atopic group compared to the psoriatic one (3.2% vs. 0%; *p*-value = 0.04).

Therefore, the former findings highlight the well-known problem of contact sensitization to methylisothiazolinone, especially in the atopic population [33]. Even with the European regulations (Cosmetic Europe and Scientific Committee on Consumer Safety) limiting the widespread use of such a preservative in cosmetic products (especially in leave-on ones), the incidence of ACD to methylisothiazolinone drastically decreased in the past years but never completely went to zero [34]. This may be due to different aspects, such as the inevitable delay of official regulations in becoming completely effective, the possible permanence of former formulations in patients’ houses, the importation of different products from non-regulated countries, and a possible change in the exposure source of the allergen (as in the case of water-based paints, household detergents, etc.) [34].

### 4.2. Contact Sensitization in AD and Psoriasis

As for the higher sensitization rate towards fragrance mix II encountered in the AD group compared to the psoriasis one, on the other hand, this finding could demonstrate a difference in terms of contact sensitization between the two underlying skin inflammatory diseases. ACD transcriptome studies on human skin demonstrated distinct polarizations of the immune response towards three of the most frequent allergens, that is to say nickel, fragrance and rubber [29] (Figure 1). According to this study, nickel may induce a Th1/Th17-driven response, whereas fragrances and rubber seem to polarize the immune system towards a Th2 cutaneous response [29]. Therefore, our findings seem to support the idea that contact sensitization to fragrance mix II could be more likely to occur in AD patients compared to psoriatic ones, as both the AD immune response is mainly Th2-driven and fragrances themselves are more prone to induce a Th2 skin immune polarization. However, some aspects seem to be in contrast with such findings. As a matter of fact, according to what was just described, AD patients should develop less PSRs towards nickel compared to psoriasis patients.

In contrast, in our study, both groups developed similar sensitization rates, as nickel is the most frequent allergen in all three examined populations (19.9% in the AD group, 15.7% in the psoriasis group and 18.6% in the control group). Moreover, although AD patients show higher sensitization rates towards fragrance mix II compared to psoriatic patients, surprisingly, no differences were found in the relative frequencies of PSRs to fragrance mix I between the two groups. As for nickel sensitization rates in both groups, this event could be at least partially explained by the idea that AD is now considered more like a complicated and heterogeneous immunological entity, with some cases, defined as “intrinsic”, of AD patients having normal IgE levels, minimum to absent barrier dysfunction and a mainly Th1-driven skin immune response [13,35,36]. Interestingly, this AD subtype seems to be more common among female patients and to predispose to metal contact sensitization [36], so that the female prevalence among the AD group in the present work (F = 250, M = 127) could have possibly influenced nickel sensitization rates. Moreover, this broader spectrum of immunological reactions in AD could at least partially justify the confusing literature data regarding the exact correlation between AD and ACD, thus possibly denying former theories according to which AD could display a protective role towards ACD only because atopic patients are not prone to develop Th1-driven skin responses [19]. Likewise, this heterogenicity of the atopic population could partially explain why other recent studies did not point out any differences between atopic and psoriatic patients in terms of both the frequency of contact sensitization and types of incriminated allergens, thus indicating the need for a better subclassification and characterization of atopic patients in such studies [37]. On the other hand, as psoriatic patients developed PSRs to fragrance mix I similarly to atopic and control patients, it possible to imagine that the different substances contained in the two fragrance mixes could have influenced such a result. Interestingly, such a difference between the two fragrance groups has already been highlighted in the literature, as Claßen et al., in 2019, demonstrated a significantly lower frequency of PSRs to fragrance mix II in the psoriasis group compared to controls, with no differences found for mix I [30].

Therefore, it is possible to suggest that each substance in both groups has its own way of polarizing the cutaneous immune response, so that fragrances in “mix I” are possibly more prone to inducing a non-Th2-driven response. Of note, more recent studies seem to mitigate the difference between fragrance mix I and II in terms of risk for ACD, as higher rates of CA to mix II have been also described among psoriasis patients [38].

Moreover, in the psoriasis group, no PSRs were found in the case of 14 tested substances out of 28 (50%), thus suggesting that psoriatic patients may be more selective in terms of developing contact sensitization compared to atopic (no PSRs only for three substances out of 28; 10.7%) and control patients (no PSRs only for one tested substance out of 28; 3.6%) [30]. This finding could indeed suggest that the psoriatic immunological background is likely to be better defined and polarized compared to the atopic one and that atopic patients could be exposed to a broader range of allergens compared to the psoriatic ones.

Therefore, it is possible to consider psoriasis as a potential “protective factor” for the development of CA (odds ratio = 0.6; *p*-value = 0.03), at least towards certain haptens, while AD could be a more generic risk factor (odds ratio: 1.42; *p*-value = 0.002) for CA.

## 5. Conclusions

The present study preliminarily demonstrates that psoriasis and AD influence the possibility of developing CA, especially towards specific allergens. As a matter of fact, although most tested substances provoked PSRs similarly in both groups, a higher rate of PSRs to fragrance mix II has been registered in the atopic group compared to the psoriatic one, according to literature data [30]. However, as nickel was the most common allergen in all examined populations, it is possible to suggest that AD could display a more heterogeneous immunological background, not exclusively Th2 driven. Therefore, the whole relationship between AD and ACD could be even more complex than previously thought.

Furthermore, the immunological background of AD and psoriasis not only seems to influence the risk of developing ACD to certain haptens but also reflects the overall selective nature of such risk. A higher number of substances in the psoriasis group did not induce any PSRs compared to the other two examined groups, so it is possible to suggest that psoriasis could display a more selective role in the development of CA, as if its immunological background is more polarized than the AD one.

In the end, psoriasis was a possible protective factor for CA, while AD seems to facilitate the development of intercurrent contact sensitizations.

The limitations of this study mainly rely upon its retrospective nature, which limited the acquisition of clinical relevance for PSR. Further studies are required to better investigate this topic and possibly better characterize the atopic population by age, sex and IgE levels.

## Figures and Tables

**Table 1:** Relative frequencies of positive skin reactions (PSRs) for each tested substance in the three examined groups (AD = atopic dermatitis, PSO = psoriasis).

Tested Substances	No. of AD Patients with PSR; Relative Frequency on Total AD Patients (%)	No. of PSO Patients with PSR; Relative Frequency on Total PSO Patients (%)	No. of Control Patients with PSR; Relative Frequency on Total Control Patients (%)
2-hydroxyethyl methacrylate	1	0	5
	(0.265)	(0.000)	(0.280)
Peru balsam	16	4	65
	(4.244)	(3.150)	(3.646)
Benzocaine	0	0	8
	(0.000)	(0.000)	(0.449)
Budesonide	2	0	3
	(0.531)	(0.000)	(0.168)
Cobalt	9	2	37
	(2.387)	(1.575)	(2.075)
Colophony	2	2	17
	(0.531)	(1.575)	(0.953)
Dimethylaminopropylamine	7	3	26
	(1.857)	(2.362)	(1.458)
Textile dye mix	8	3	43
	(2.122)	(2.362)	(2.412)
N-Isopropyl-N-phenyl-*p*-phenylenediamine	2	0	5
	(0.531)	(0.000	(0.280)
Formaldehyde	6	0	18
	(1.592)	(0.000)	(1.010)
Hydrocortisone	0	0	4
	(0.000)	(0.000)	(0.224)
Kathon CG	16	2	53
	(4.244)	(1.575)	(2.973)
Lanolin	4	0	12
	(1.061)	(0.000)	(0.673)
Lyral	2	0	12
	(0.531)	(0.000)	(0.673)
Mercaptobenzothiazole	1	0	6
	(0.265)	(0.000)	(0.337)
Mercapto mix	2	0	2
	(0.531)	(0.000)	(0.112)
Methylisothiazolinone	16	4	40
	(4.244)	(3.150)	(2.243)
Neomycin	5	0	8
	(1.326)	(0.000)	(0.449)
Nickel sulphate	75	20	331
	(19.894)	(15.748)	(18.564)
Paraben mix	1	0	0
	(0.265)	(0.000)	(0.000)
*p*-Phenylenediamine	4	3	42
	(1.061)	(2.362)	(2.356)
Potassium dichromate	11	4	41
	(2.918)	(3.150)	(2.299)
Fragrance mix I	11	2	56
	(2.918)	(1.575)	(3.141)
Fragrance mix II	12	0	33
	(3.183)	(0.000)	(1.851)
Epoxy resin	1	0	1
	(0.265)	(0.000)	(0.056)
*p*-tert-butylphenol-formaldehyde resin	1	1	5
	(0.265)	(0.787)	(0.280)
Sorbitan sesquioleate	0	1	2
	(0.000)	(0.787)	(0.112)
Thiuram mix	5	2	19
	(1.326)	(1.575)	(1.066)
***TOTAL***	***220***	***53***	***894***
	***(58.35)***	***(41.730)***	***(50.140)***

**Table 2:** Relative frequencies of PSRs between the AD group and the control group (statistically significant *p*-values are reported in bold italics and marked with “*”).

Tested Substances	AD Relative Frequency in Total AD Patients (%)	CONTROLS Relative Frequency in Total Control Patients (%)	Two-Sample Proportion “z” Test (*p*-Value)
2-hydroxyethyl methacrylate	0.265	0.280	0.959437
Peru balsam	4.244	3.646	0.578398
Benzocaine	0.000	0.449	0.192573
Budesonide	0.531	0.168	0.1836
Cobalt	2.387	2.075	0.702923
Colophony	0.531	0.953	0.424263
Dimethylaminopropylamine	1.857	1.458	0.566504
Textile dye mix	2.122	2.412	0.736472
N-Isopropyl-N-phenyl-*p*-phenylenediamine	0.531	0.280	0.437627
Formaldehyde	1.592	1.010	0.327368
Hydrocortisone	0.000	0.224	0.357307
Kathon CG	4.244	2.973	0.20212
Lanolin	1.061	0.673	0.42475
Lyral	0.531	0.673	0.754049
Mercaptobenzothiazole	0.265	0.337	0.824953
Mercapto mix	0.531	0.112	0.0860723
**Methylisothiazolinone**	**4.244**	2.243	0.0263604 *
**Neomycin**	**1.326**	0.449	0.0453308 *
Nickel sulphate	19.894	18.564	0.548237
**Paraben mix**	**0.265**	0.000	0.0296127 *
*p*-Phenylenediamine	1.061	2.356	0.113681
Potassium dichromate	2.918	2.299	0.47673
Fragrance mix I	2.918	3.141	0.820491
Fragrance mix II	3.183	1.851	0.0998771
Epoxy resin	0.265	0.056	0.225061
*p*-tert-butylphenol-formaldehyde resin	0.265	0.280	0.959437
Sorbitan sesquioleate	0.000	0.112	0.515308
Thiuram mix	1.326	1.066	0.660922
**TOTAL**	**58.35**	**50.14**	0.00373272 *

**Table 3:** Relative frequencies of PSR between the AD group and the PSO group (statistically significant *p*-values are reported in bold italics and marked with “*”).

Tested Substances	AD Relative Frequency in Total AD Patients (%)	PSO Relative Frequency in Total PSO Patients (%)	Two-Sample Proportion “z” Test (*p*-Value)
2-hydroxyethyl methacrylate	0.265	0.000	0.561253
Peru balsam	4.244	3.150	0.584769
Benzocaine	0.000	0.000	///////
Budesonide	0.531	0.000	0.410821
Cobalt	2.387	1.575	0.587842
Colophony	0.531	1.575	0.251344
Dimethylaminopropylamine	1.857	2.362	0.723893
Textile dye mix	2.122	2.362	0.872707
N-Isopropyl-N-phenyl-*p*-phenylenediamine	0.531	0.000	0.410821
Formaldehyde	1.592	0.000	0.15265
Hydrocortisone	0.000	0.000	///////
Kathon CG	4.244	1.575	0.160943
Lanolin	1.061	0.000	0.243839
Lyral	0.531	0.000	0.410821
Mercaptobenzothiazole	0.265	0.000	0.561253
Mercapto mix	0.531	0.000	0.410821
Methylisothiazolinone	4.244	3.150	0.160943
Neomycin	1.326	0.000	0.192128
Nickel sulphate	19.894	15.748	0.301515
Paraben mix	0.265	0.000	0.561253
*p*-Phenylenediamine	1.061	2.362	0.278504
Potassium dichromate	2.918	3.150	0.894214
Fragrance mix I	2.918	1.575	0.408954
**Fragrance mix II**	**3.183**	**0.000**	0.0418555 *
Epoxy resin	0.265	0.000	0.561253
*p*-tert-butylphenol-formaldehyde resin	0.265	0.787	0.418229
Sorbitan sesquioleate	0.000	0.787	0.0845912
Thiuram mix	1.326	1.575	0.0680477
**TOTAL**	**58.35**	**41.73**	0.00830354 *

**Table 4:** Relative frequencies of PSRs between the PSO group and the control group (statistically significant *p*-values are reported in bold italics and marked with “*”).

Tested Substances	PSO Relative Frequency in Total PSO Patients (%)	CONTROLS Relative Frequency in Total Control Patients (%)	Two-Sample Proportion “z” Test (*p*-Value)
2-hydroxyethyl methacrylate	0.000	0.280	0.550135
Peru balsam	3.150	3.646	0.772291
Benzocaine	0.000	0.449	0.449377
Budesonide	0.000	0.168	0.643634
Cobalt	1.575	2.075	0.70008
Colophony	1.575	0.953	0.495411
Dimethylaminopropylamine	2.362	1.458	0.420854
Textile dye mix	2.362	2.412	0.971978
N-Isopropyl-N-phenyl-*p*-phenylenediamine	0.000	0.280	0.550135
Formaldehyde	0.000	1.010	0.255256
Hydrocortisone	0.000	0.224	0.593112
Kathon CG	1.575	2.973	0.362802
Lanolin	0.000	0.673	0.353699
Lyral	0.000	0.673	0.353699
Mercaptobenzothiazole	0.000	0.337	0.512619
Mercapto mix	0.000	0.112	0.705704
Methylisothiazolinone	3.150	2.243	0.510725
Neomycin	0.000	0.449	0.449377
Nickel sulphate	15.748	18.564	0.428516
Paraben mix	0.000	0.000	///////
*p*-Phenylenediamine	2.362	2.356	0.996206
Potassium dichromate	3.150	2.299	0.541681
Fragrance mix I	1.575	3.141	0.320382
Fragrance mix II	0.000	1.851	0.121969
Epoxy resin	0.000	0.056	0.789503
*p*-tert-butylphenol-formaldehyde resin	0.787	0.280	0.323916
Sorbitan sesquioleate	0.787	0.112	0.0633728
Thiuram mix	1.575	1.066	0.594954
TOTAL	41.73	50.14	0.0670963

## Data Availability

The data that support the findings of this study are available from the corresponding author upon reasonable request.

## Supplementary Materials

The following supporting information can be downloaded at: https://www.mdpi.com/article/10.3390/diagnostics15060766/s1, Table S1: Tested haptens included in the present study, according to S.I.D.A.P.A. standard series (years 2016–2022).

## Abbreviations

The following abbreviations are used in this manuscript:

CD	contact dermatitis
ICD	irritant contact dermatitis
ACD	allergic contact dermatitis
TNF-α	Tumor necrosis factor alpha
APCs	antigen-presenting cells
Th	T helper
IFN-γ	interferon gamma
AD	atopic dermatitis
SOC	skin of color
Tc	T cytotoxic
CA	contact allergy
S.I.D.A.P.A	Società Italiana di Dermatologia Allergologica Professionale e Ambientale
PSR	positive skin reaction
D	day
