# Supplementary material for: Contact Allergy in Atopic Dermatitis and Psoriasis: A Retrospective Study
Source: Diagnostics (Basel). 2025 Mar 19;15(6):766. doi: 10.3390/diagnostics15060766 (PMC11940888; doi:10.3390/diagnostics15060766)
Supplement: Supplementary file 1 [file diagnostics-15-00766-s001.zip › diagnostics-3522549-supplementary.pdf]

Supplementary material

Table S1: tested haptens included in the present study, according to S.I.D.A.P.A. standard series (years 2016-2022)

S.I.D.A.P.A. standard series (2016-2022)
2-hydroxyethyl methacrylate 2% pet.
Peru Balsam 25% pet.
Benzocaine 5% pet.
Budesonide 0.01% pet.
Cobalt 1% pet.
Colophony 20% pet.
Dimethylaminopropylamine 1% aq.
Textile dye mix 6.6% pet.
N-Isopropyl-N-phenyl-p-phenylenediamine 0.1% pet.
Formaldehyde 2% aq.
Hydrocortisone 1% pet.
Kathon CG 0.02% aq.
Lanolin 30% pet.
Lyral 5% pet.
Mercaptobenzothiazole 2% pet.
Mercapto mix 2% pet.
Methylisothiazolinone 0.2% aq.
Neomycin 20% pet.
Nickel sulphate 5% pet.
Paraben mix 16% pet.
p-Phenylenediamine 1% pet.
Potassium dichromate 0.5% pet.
Fragrance mix I 8% pet.
Fragrance mix II 14% pet.
Epoxy resin 1% pet.
p-tert-butylphenol-formaldehyde resin
Sorbitan sesquioleate 20% pet.
Thiuram mix 1% pet.
